# ATF2-Induced lncRNA GAS8-AS1 Promotes Autophagy of Thyroid Cancer Cells by Targeting the miR-187-3p/ATG5 and miR-1343-3p/ATG7 Axes

**DOI:** 10.1016/j.omtn.2020.09.022

**Published:** 2020-09-23

**Authors:** Yuan Qin, Wei Sun, Zhihong Wang, Wenwu Dong, Liang He, Ting Zhang, Liang Shao, Hao Zhang

**Affiliations:** 1 Department of Thyroid Surgery, The First Hospital of China Medical University, Shenyang, Liaoning 110001, P. R. China

**Keywords:** GAS8-AS1, lncRNA, thyroid cancer, autophagy, transcription factor

## Abstract

Long non-coding RNAs (lncRNAs) play an essential regulatory role in multiple cancers. However, the role of lncRNAs in papillary thyroid carcinoma (PTC) is still unknown. Here, GAS8-AS1, a novel lncRNA that is significantly downregulated in PTC, was selected for further investigation. The roles of GAS8-AS1 in PTC cells were verified by gain- and loss-of-function experiments. The functional mechanism of GAS8-AS1 on the microRNA (miR)-187-3p/ATG5 axis and miR-1343-3p/ATG7 axis in PTC cells was evaluated using bioinformatics analysis, luciferase reporter assay, Cell Counting Kit-8 (CCK-8) assay, immunohistochemistry analysis, transmission electron microscopy, and immunofluorescence. We found that GAS8-AS1 was downregulated in PTC tissues and cell lines. In patients with PTC, low GAS8-AS1 expression was associated with higher tumor-node-metastasis (TNM) stage and lymph node metastasis (LNM). Functionally, GAS8-AS1 significantly promoted autophagy and inhibited PTC cell proliferation *in vitro* and promoted tumorigenesis *in vivo*. Mechanistically, GAS8-AS1 acted as a sponge of miR-187-3p and miR-1343-3p and upregulated ATG5 and ATG7 expression, respectively. The transcription factor ATF2 regulated GAS8-AS1 by binding to the GAS8-AS1 promoter. In conclusion, upregulation of ATF2 activated GAS8-AS1–promoted autophagy of PTC cells by sponging oncogenic miR-187-3p and miR-1343-3p and upregulating the expression of ATG5 and ATG7, respectively, making GAS8-AS1 a potential prognostic biomarker and therapeutic target for PTC.

## Graphical Abstract

**Figure:**
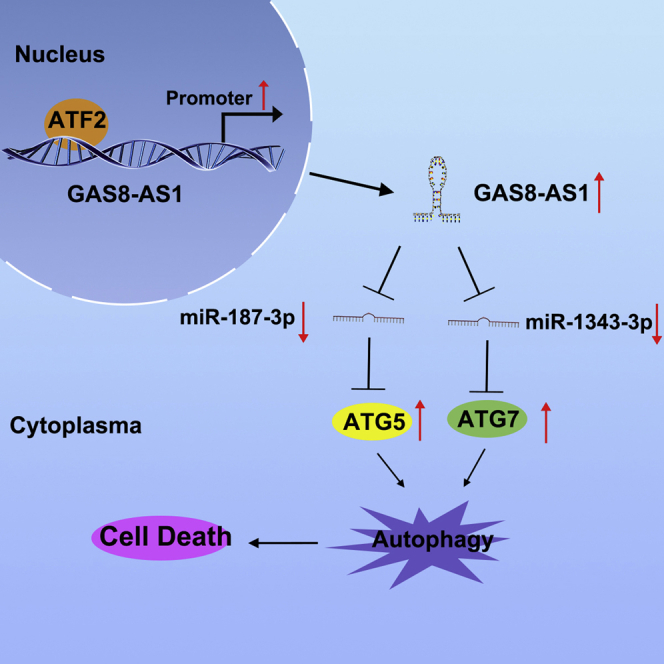

## Teaser

Zhang and colleagues demonstrated that GAS8-AS1 was downregulated in PTC tissue and cells and was related to LNM and TNM stages in PTC patients. Mechanistically, ATF2 activated GAS8-AS1–promoted autophagy of PTC cells by sponging oncogenic miR-187-3p and miR-1343-3p and upregulating the ATG5 and ATG7, respectively.

## Introduction

Thyroid cancer is the most common endocrine-related malignant tumor, and its occurrence per year has more than doubled in the past two decades. The latest epidemiological statistics state that the global incidence of thyroid cancer is ninth overall and fifth in women.^1^ Most of this growth is attributed to papillary thyroid carcinoma (PTC), which constitutes >80% of all thyroid cancer cases.^2^^,^^3^ Currently, the most common treatments for PTC include surgery and radioactive iodine therapy, and the 5-year survival rate is >95%. Despite the good prognosis, some patients still have untreatable invasion, local metastasis, and tumor recurrence, which results in the limitations of the aforementioned treatments.^3^ Such patients have poor prognosis, and the 10-year survival rate drops to 40%.^4^ In addition, 10%–15% of patients with PTC experience relapse and metastasis after therapy.^5^ Therefore, elucidating the molecular mechanisms of PTC development and establishing promising prognostic factors for PTC treatment is crucial.

Long non-coding RNAs (lncRNAs) have attracted widespread attention due to their potential roles in development and disease, including cancer.^6^ lncRNAs comprise a heterogeneous family of RNA molecules of >200 nucleotides (nt) with no or limited protein-coding potential. Aberrant lncRNA expression has been observed in various cancers.^7^ An increasing number of lncRNAs have recently been reported to play an important role in PTC carcinogenesis and development.^8^^,^^9^ The lncRNA GAS8 antisense RNA 1 (GAS8-AS1) is located in intron 2 of GAS8 and transcribes a 994-nt ncRNA in the opposite orientation of GAS8.^10^ Recent reports have suggested that GAS8-AS1 contributes to anti-tumorigenesis in some cancers, such as colorectal cancer,^11^ hepatocellular cancer,^12^ thyroid cancer,^13^ and osteosarcoma.^14^

Autophagy captures and degrades intracellular components in lysosomes, thereby sustaining metabolic homeostasis and promoting the growth of a broad spectrum of cancers under certain conditions. Autophagy can be divided into at least three types according to the physiological role and the pattern of cargo delivery to the lysosome: chaperone-mediated autophagy, microautophagy, and macroautophagy. Macroautophagy, the main catabolic mechanism, is modulated by a limited number of autophagy-related genes (ATGs).^15^^,^^16^ Recent studies have discussed autophagy and its molecular mechanism in carcinomas in depth,^17^, ^18^, ^19^ and the role of ATG5 and ATG7 in this process has gained more attention. ATG5, located on chromosome 6, is essential for autophagosome formation and autophagy promotion.^20^ ATG7 encodes an E1-like ligase, which is essential for forming a mature autophagosomal membrane.^21^ Although the detailed role of autophagy in thyroid cancer pathogenesis has not been elucidated, recent studies have shed light on the underlying mechanisms of autophagy in regulating thyroid cancer development and dedifferentiation.^22^, ^23^, ^24^

Previously, we showed that GAS8-AS1 inhibits cell proliferation through ATG5-mediated autophagy in PTC.^25^ However, its specific regulatory mechanism is still unclear. In the present study, we determined that GAS8-AS1 was stably downregulated in PTC tissue samples and cell lines and analyzed the potential relationship between GAS8-AS1 levels and PTC clinicopathological features. Mechanistically, the transcription factor (TF) ATF2 activated GAS8-AS1–promoted autophagy of PTC cells by sponging oncogenic microRNA (miR)-187-3p and miR-1343-3p and upregulating the expression levels of ATG5 and ATG7, respectively. Therefore, GAS8-AS1 could be a promising biomarker and therapeutic target for patients with PTC.

## Results

### Downregulation of GAS8-AS1 Was Associated with Higher TNM Stage and LNM in Patients with PTC and Mainly Localized in PTC Cytoplasm

**Figure 1:**
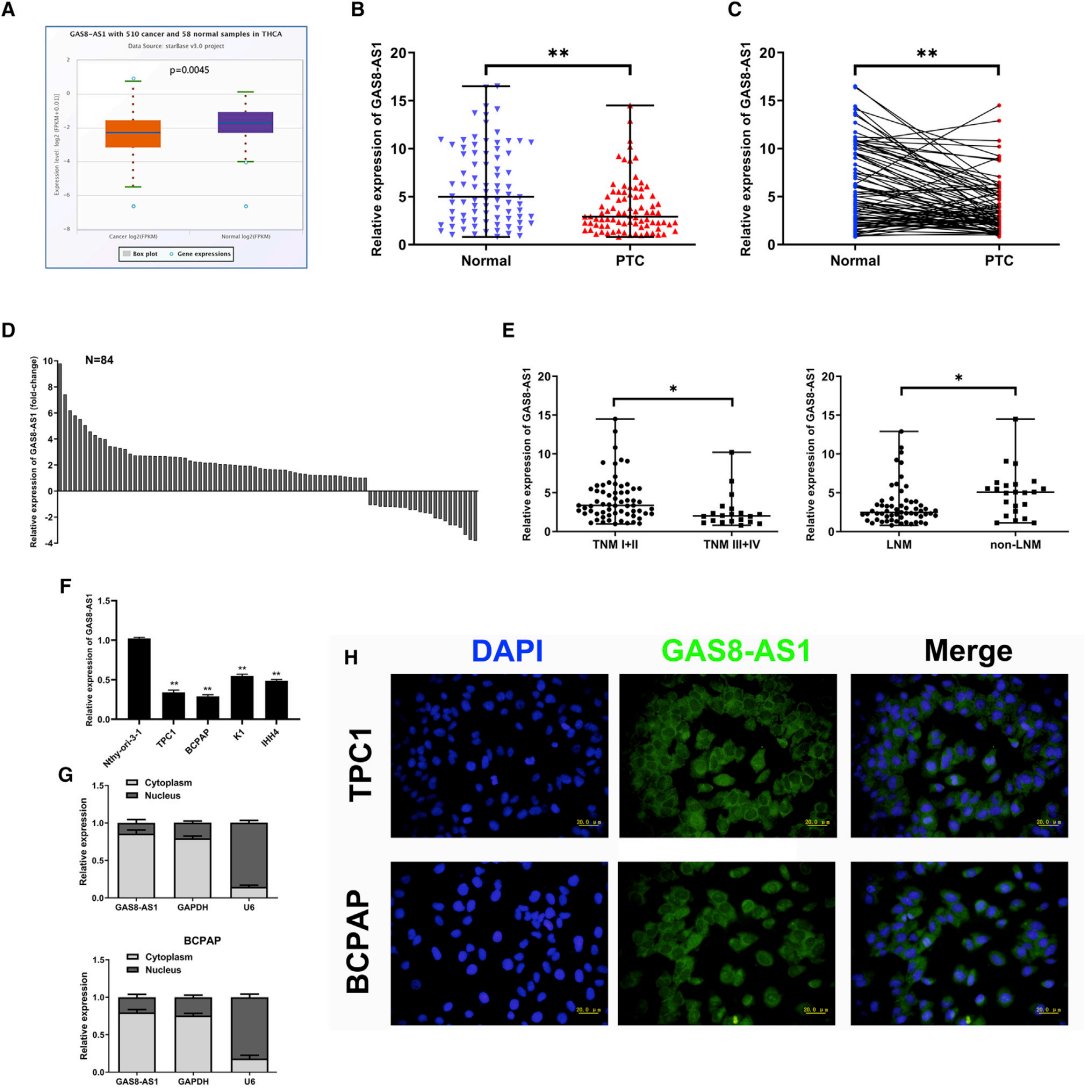
GAS8-AS1 Was Downregulated and Associated with Higher TNM Stage and LNM, Mainly Localizing in PTC Cytoplasm (A) GAS8-AS1 expression in PTC and normal tissues from TCGA database. (B) The relative expression of GAS8-AS1 in 84 pairs of PTC tissues and adjacent non-cancerous tissues. Statistical differences were analyzed using the Wilcoxon signed-rank test; data are shown as the median and range. (C and D) The comparison (C) and fold change (D) of GAS8-AS1 expression between PTC tissues and the corresponding adjacent non-cancerous tissues. (E) The relative expression levels of GAS8-AS1 in TNM I+II and TNM III+IV or LNM and non-LNM. Statistical differences were analyzed using the Wilcoxon signed-rank test; data are shown as the median and range. (F) Relative expression of GAS8-AS1 in PTC cells and the normal thyroid cells was measured by qRT-PCR. (G) Nuclear and cytoplasmic RNA fractions were isolated from PTC cells. GAS8-AS1 was located mainly in the cytoplasm; GAPDH and U6 were used as controls. (H) FISH verification that GAS8-AS1 was localized mainly in the cytoplasm. Scale bar, 20 μm. Statistical differences were analyzed using the independent samples t test; data are shown as the mean ± standard error of the mean based on three independent experiments. ∗p < 0.05, ∗∗p < 0.01.

We predicted a significant increase in GAS8-AS1 expression in normal tissues using The Cancer Genome Atlas (TCGA) database (Figure 1A). Subsequently, the qRT-PCR results showed that the GAS8-AS1 expression levels in 84 PTC tissues were lower than those in the paired adjacent non-cancerous tissues (Figures 1B and 1C). GAS8-AS1 was increased in 62/84 (73.8%) of the adjacent non-cancerous tissues compared with the paired PTC tissues (Figure 1D). Additionally, clinical data demonstrated that low GAS8-AS1 expression was correlated with higher tumor-node-metastasis (TNM) stage (III/IV) and lymph node metastasis (LNM) in patients with PTC (Table 1). The qRT-PCR results also showed that GAS8-AS1 expression was notably lower in patients with higher TNM stage and LNM (Figure 1E). Consistent with the PTC tissue sample data, GAS8-AS1 expression was markedly lower in PTC cell lines (TPC1, BCPAP, K1, IHH4) than in the normal thyroid cell line Nthy-ori-3-1 (Figure 1F). Subcellular fractionation and fluorescence *in situ* hybridization (FISH) analysis revealed that GAS8-AS1 was predominantly located in the cytoplasm of TPC1 and BCPAP cells (Figures 1G and 1H). These results indicate that GAS8-AS1 levels are typically reduced in PTC tissues and cell lines, suggesting that it may be involved in PTC progression.

**Table 1:** Correlation Between GAS8-AS1 Expression and Clinicopathological Features in PTC Tissues (n = 84)

Characteristic	n	High Expression (%)	Low Expression (%)	p Value
Gender				
Male	29	13 (44.8)	16 (55.2)	0.831
Female	55	26 (47.3)	29 (52.7)	
Age, years				
<55	57	28 (49.1)	29 (50.9)	0.472
≥55	27	11 (40.7)	16 (59.3)	
Extrathyroidal extension				
Yes	26	13 (50)	13 (50)	0.66
No	58	26 (44.8)	32 (55.2)	
TNM staging				
I–II	64	34 (53.1)	30 (46.9)	0.027∗
III–IV	20	5 (25)	15 (75)	
LNM				
Yes	61	22 (36.1)	39 (63.9)	0.002∗
No	23	17 (73.9)	6 (26.1)	
Multifocality				
Yes	24	11 (45.8)	13 (54.2)	0.945
No	60	28 (46.7)	32 (53.3)	
Tumor size, cm				
<2	48	23 (47.9)	25 (52.1)	0.752
≥2	36	16 (44.4)	20 (55.6)	
Hashimoto thyroiditis				
Yes	19	8 (42.1)	11 (57.9)	0.668
No	65	31 (47.7)	34 (52.3)	

∗ p < 0.05.

### GAS8-AS1 Promoted ATG5 and ATG7 Expression in PTC Cells

**Figure 2:**
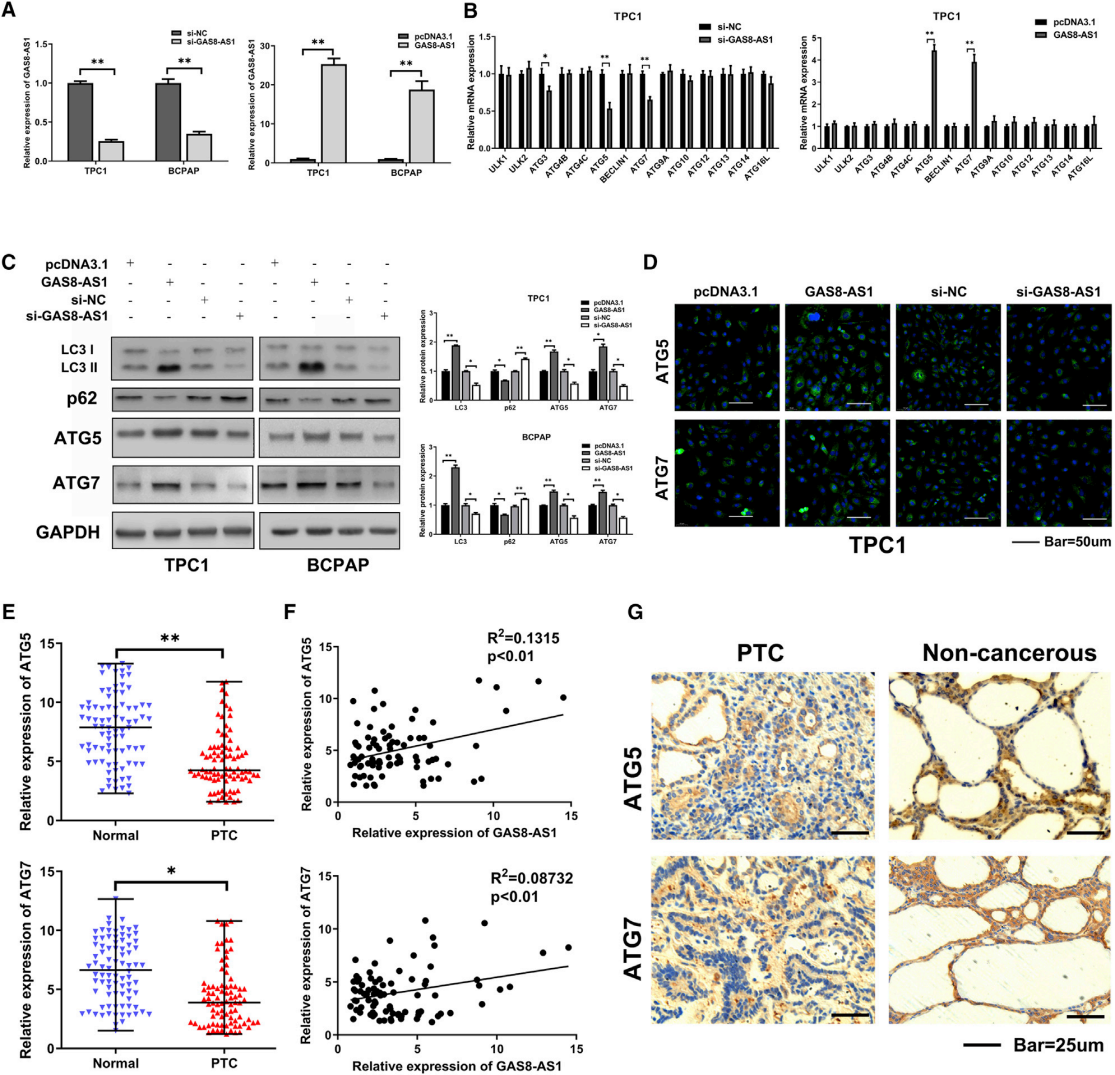
GAS8-AS1 Promoted ATG5 and ATG7 Expression in PTC Cells (A) qRT-PCR analysis confirmed that GAS8-AS1 was successfully overexpressed and knocked down in TPC1 and BCPAP cells. (B) The relative expression level of related ATGs after downregulated and overexpressed GAS8-AS1 was detected by qRT-PCR in TPC1 cells. (C) Western blotting detection of the autophagy-relevant proteins (LC3, p62, ATG5, ATG7) after transfection of si-GAS8-AS1 and pcDNA3.1-GAS8-AS1 in TPC1 and BCPAP cells. (D) IF evaluation of ATG5 and ATG7 levels during GAS8-AS1 overexpression and knockdown in TPC1 cells. Scale bar, 50 μm. (E) qRT-PCR measurement of the relative expression levels of ATG5 and ATG7 in 84 pairs of PTC tissues and adjacent non-cancerous tissues. Statistical differences were analyzed using the Wilcoxon signed-rank test; data are shown as the median and range. (F) The correlations between GAS8-AS1 and ATG5, ATG7 in PTC tissues were analyzed using Pearson’s correlation. (G) Representative ATG5 and ATG7 IHC staining of PTC tissues compared to paired normal thyroid tissues. Scale bar, 25 μm. Statistical differences were analyzed using the independent samples t test; data are shown as the mean ± standard error of the mean based on three independent experiments. ∗p < 0.05, ∗∗p < 0.01.

We confirmed in a previous article that GAS8-AS1 can activate autophagy in PTC cells.^25^ To further explore the biological role of GAS8-AS1, TPC1 and BCPAP cells were transfected with GAS8-AS1 overexpression vector (pcDNA3.1-GAS8-AS1) and with small interfering RNA (siRNA) targeting GAS8-AS1 (si-GAS8-AS1), then the overexpression and silencing efficiency of GAS8-AS1 were detected by qRT-PCR. As expected, GAS8-AS1 expression was higher in the pcDNA3.1-GAS8-AS1 group than in the control vector (pcDNA3.1) group and was less than 50% in the si-GAS8-AS1 group as compared with the non-silencing control (si-NC) group (Figure 2A). ATGs play a key role in the multistep catabolic processes of autophagy. To reveal the mechanism of GAS8-AS1–mediated autophagy activation, we examined the mRNA expression of several ATGs by qRT-PCR. In the TPC1 cells, silencing GAS8-AS1 downregulated ATG3, ATG5, and ATG7, while GAS8-AS1 overexpression upregulated ATG5 and ATG7 markedly (Figure 2B). The BCPAP cell line yielded similar results (Figure S1). Western blotting confirmed that amplifying GAS8-AS1 expression increased LC3II/LC3I, ATG5, and ATG7 protein expression and decreased that of p62; the opposite result was obtained when GAS8-AS1 expression was decreased (Figure 2C). The punctate aggregates of ATG5 and ATG7 staining increased with increased GAS8-AS1 expression, and the opposite was also true (Figure 2D). The relative expression levels of ATG5 and ATG7 were verified by qRT-PCR and immunohistochemistry (IHC) in 84 pairs of PTC tissues and matched adjacent non-cancerous tissues. Figures 2E and 2G show that the PTC tissues had significantly downregulated ATG5 and ATG7 expression compared with the matched adjacent non-cancerous tissues at the mRNA and protein levels (Table 2). Spearman’s correlation analysis showed a positive relationship between GAS8-AS1 and ATG5, ATG7 in PTC tissue (Figure 2F).

**Table 2:** IHC Analysis of ATG5 and ATG7 Protein Levels in 84 Paired PTC Tissues and Adjacent Non-Cancerous Tissues

Sample	ATG5		p Value	ATG7		p Value
	+	−		+	−	
PTC tissues	30	54	0.005∗	37	47	0.013∗
Adjacent non-cancerous tissues	48	36		53	31	

∗ p < 0.05.

### miR-187-3p and miR-1343-3p Targeting Inhibited ATG5 and ATG7 Expression, Respectively

**Figure 3:**
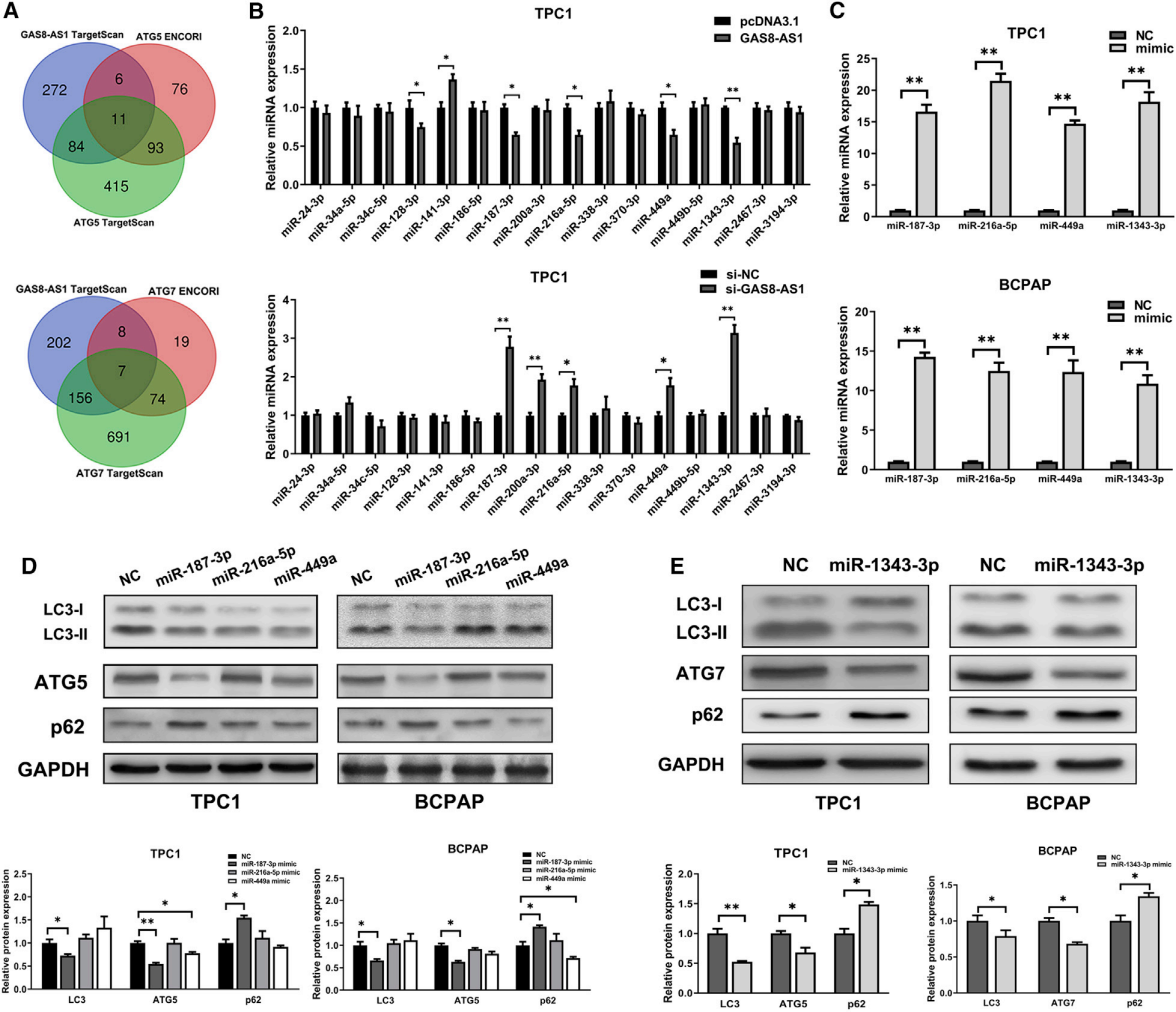
miR-187-3p and miR-1343-3p Targeting Inhibited ATG5 and ATG7 Expression in PTC Cells, Respectively (A) The miRNAs potentially targeting GAS8-AS1, ATG5, and ATG7 were predicted using bioinformatics software. (B) qRT-PCR measurement of the relative expression of the predicted miRNAs after si-GAS8-AS1 or pcDNA3.1-GAS8-AS1 transfection in TPC1 cells. (C) qRT-PCR analysis of overexpression efficiency after miR-187-3p, miR-216a-5p, miR-449a, and miR-1343-3p mimic transfection in PTC cells. (D) Western blotting detection of the autophagy-relevant proteins (LC3, ATG5) after miR-187-3p, miR-216a-5p, and miR-449a mimic transfection in PTC cells. (E) Western blotting detection of the autophagy-relevant proteins (LC3, ATG7) after miR-1343-3p mimic transfection in PTC cells. Statistical differences were analyzed using the independent samples t test; data are shown as the mean ± standard error of the mean based on three independent experiments. ∗p < 0.05, ∗∗p < 0.01.

lncRNA subcellular localization is closely related to their biological function and potential molecular roles. As an important lncRNA regulatory mechanism, the competing endogenous RNA (ceRNA) theory argues that lncRNAs, which are highly expressed in the cytoplasm, can bind competitively to miRNAs and regulate the downstream target genes. Therefore, GAS8-AS1 was speculated to regulate PTC cell function through the ceRNA mechanism. The TargetScanHuman 7.2 (http://www.targetscan.org/vert_72/) and ENCORI (http://starbase.sysu.edu.cn/) databases revealed 16 miRNAs that may bind with GAS8-AS1 and ATG5, ATG7 via complementary base pairing (Figure 3A). Next, the expression of the candidate miRNAs was detected by qRT-PCR in the downregulation and overexpression of GAS8-AS1 in the TPC1 (Figure 3B) and BCPAP cells (Figure S2), respectively. Among the candidate miRNAs, miR-187-3p, miR-216a-5p, and miR-449a may regulate ATG5 expression, while miR-1343-3p may regulate ATG7 expression. To confirm this, we overexpressed candidate miRNAs (Figure 3C) and detected LC3II/LC3I, ATG5, ATG7, and p62 expression in PTC cells. Western blotting showed that only miR-187-3p inhibited ATG5 expression, and miR-1343-3p inhibited ATG7 expression, indicating that ATG5 and ATG7 may be target genes of miR-187-3p and miR-1343-3p, respectively. Interestingly, miR-187-3p and miR-1343-3p both inhibited the LC3II/LC3I rate and promoted p62 expression, and we speculated that they could also be related to PTC autophagy (Figures 3D and 3E).

### Knockdown of miR-187-3p and miR-1343-3p Inhibited PTC Cell Proliferation but Promoted Autophagy

**Figure 4:**
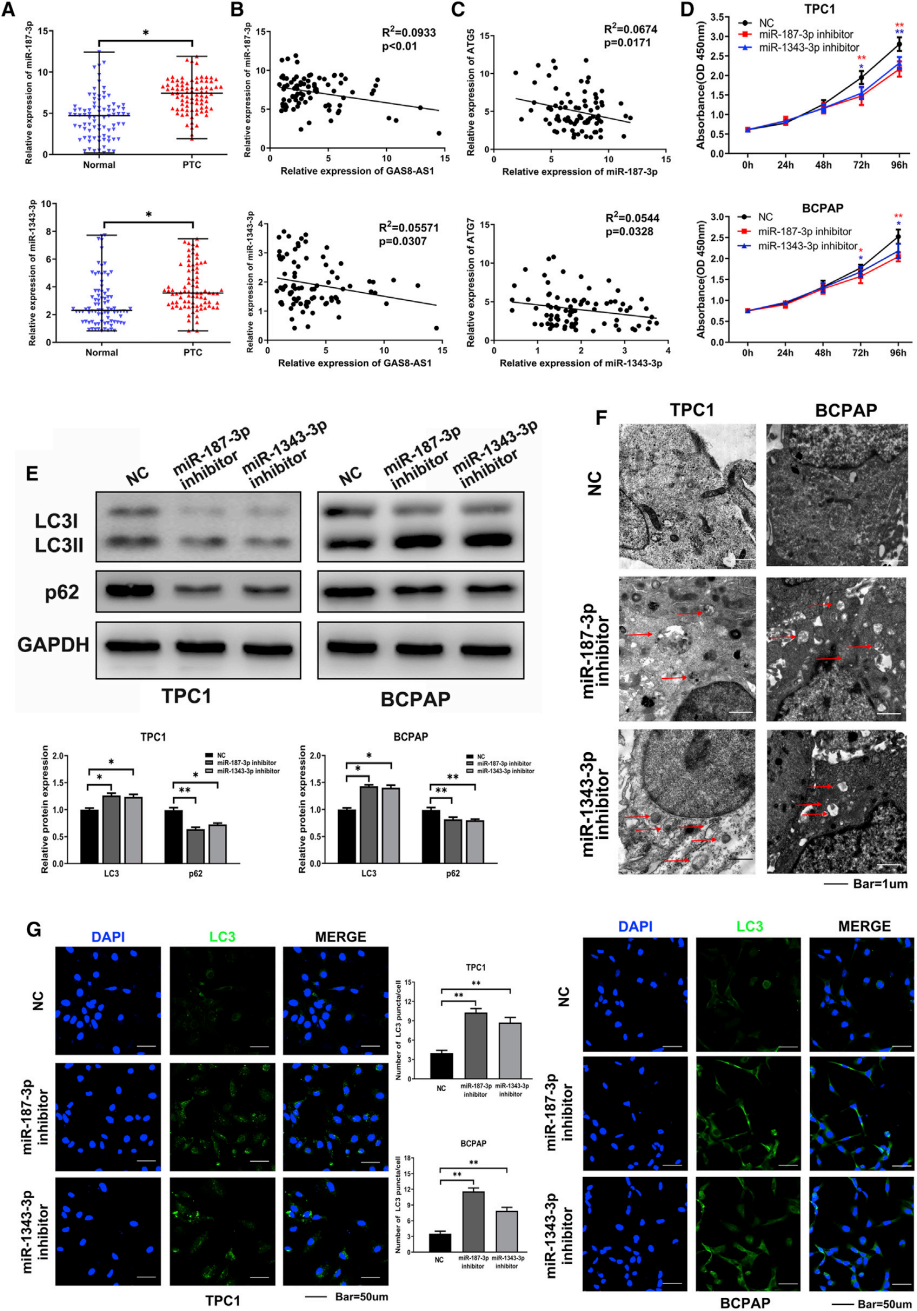
Knockdown of miR-187-3p and miR-1343-3p Inhibited PTC Cell Proliferation but Promoted Autophagy (A) qRT-PCR determination of the relative expression of miR-187-3p and miR-1343-3p in 84 pairs of PTC tissues and adjacent non-cancerous tissues. Statistical differences were analyzed using the Wilcoxon signed-rank test; data are shown as the median and range. (B) The correlations between GAS8-AS1 and miR-187-3p and miR-1343-3p in PTC tissues were analyzed using Pearson’s correlation. (C) The correlations between miR-187-3p and ATG5 and miR-1343-3p and ATG7 in PTC tissues were analyzed using Pearson’s correlation. (D–G) Growth was evaluated using CCK-8 (D), autophagy-relevant proteins (LC3, p62) were detected by western blotting (E), autophagosomes were shown using TEM (red arrows) (F), and LC3 puncta were evaluated using IF (G) after miR-187-3p inhibitor and miR-1343-3p inhibitor transfection in TPC1 and BCPAP cells. Statistical differences were analyzed using the independent samples t test; data are shown as the mean ± standard error of the mean based on three independent experiments. ∗p < 0.05, ∗∗p < 0.01.

Next we studied the expression and function of miR-187-3p and miR-1343-3p. qRT-PCR showed higher expression of both miR-187-3p and miR-1343-3p in the 84 PTC tissues compared with the paired adjacent non-cancerous tissues (Figure 4A). In the PTC tissues, miR-187-3p was negatively correlated with GAS8-AS1 and ATG5, and miR-1343-3p was negatively correlated with GAS8-AS1 and ATG7 (Figures 4B and 4C). To verify the effect of miR-187-3p and miR-1343-3p on cell proliferation and autophagy, PTC cells with low miR-187-3p and miR-1343-3p expression were constructed (Figure S3). In PTC cells, Cell Counting Kit-8 (CCK-8) assays showed that knockdown of miR-187-3p and miR-1343-3p inhibited proliferation (Figure 4D); western blotting showed that miR-187-3p and miR-1343-3p knockdown promoted LC3II/LC3I expression and decreased p62 expression (Figure 4E). Transmission electron microscopy (TEM) and immunofluorescence (IF) showed more autophagosomes and punctate aggregates of LC3 staining when miR-187-3p and miR-1343-3p were downregulated in PTC cells (Figures 4F and 4G). The results indicate that the knockdown of miR-187-3p and miR-1343-3p was the driving force in activating PTC cell autophagy.

### miR-187-3p and miR-1343-3p Overexpression Partly Impaired GAS8-AS1–Induced PTC Cell Autophagy and Inhibition of Proliferation

**Figure 5:**
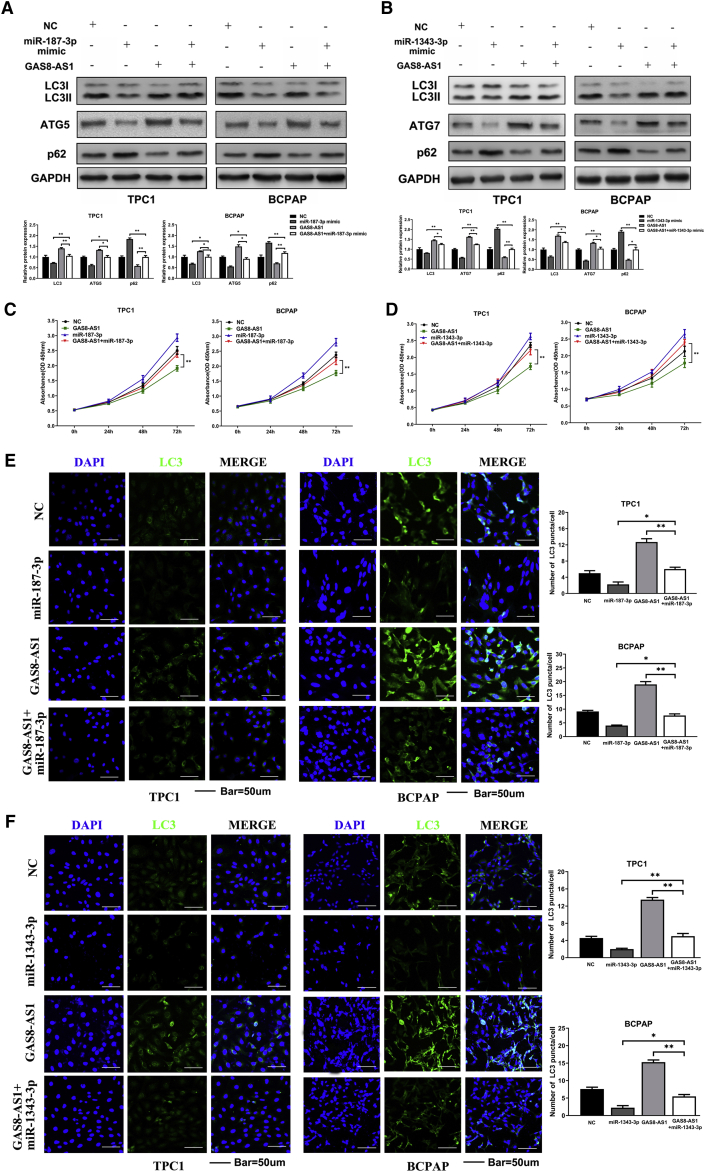
miR-187-3p and miR-1343-3p Overexpression Partly Impaired GAS8-AS1–Induced Inhibition of Malignant Behavior in PTC Cells (A–C) LC3II/LC3I, ATG5, and p62 levels were analyzed using western blotting (A), cell growth was analyzed using CCK-8 assay (B), and LC3 puncta were evaluated using IF (C) in TPC1 and BCPAP cells transfected with NC, miR-187-3p mimic, GAS8-AS1, or co-transfected GAS8-AS1 and miR-187-3p mimic. (D–F) LC3II/LC3I, ATG7, and p62 levels were analyzed using western blotting (D), cell growth was analyzed using CCK-8 (E), and LC3 puncta were evaluated using IF (F) in TPC1 and BCPAP cells transfected with NC, miR-1343-3p mimic, GAS8-AS1, or co-transfected with GAS8-AS1 and miR-1343-3p mimic. Statistical differences were analyzed using the independent samples t test; data are shown as the mean ± standard error of the mean based on three independent experiments. ∗p < 0.05, ∗∗p < 0.01.

The rescue experiments confirmed that miR-187-3p and miR-1343-3p can restore the effect of GAS8-AS1 on PTC cell proliferation and autophagy. Western blotting revealed that miR-187-3p mimics significantly reversed the autophagy effect of GAS8-AS1, which promoted LC3II/LC3I and ATG5 expression, and inhibited p62 expression (Figure 5A). Similarly, miR-1343-3p overexpression impaired GAS8-AS1–induced upregulation of LC3II/LC3I and ATG7 expression and the inhibition of p62 expression (Figure 5B). CCK-8 assays showed that miR-187-3p and miR-1343-3p could weaken the proliferation inhibition of GAS8-AS1 in TPC1 and BCPAP cells (Figures 5C and 5D). Additionally, miR-187-3p and miR-1343-3p also antagonized the increased punctate aggregates of LC3 staining induced by GAS8-AS1 (Figures 5E and 5F).

### GAS8-AS1 Acts as a ceRNA in Regulating ATG5 and ATG7 Expression by Binding to miR-187-3p and miR-1343-3p

**Figure 6:**
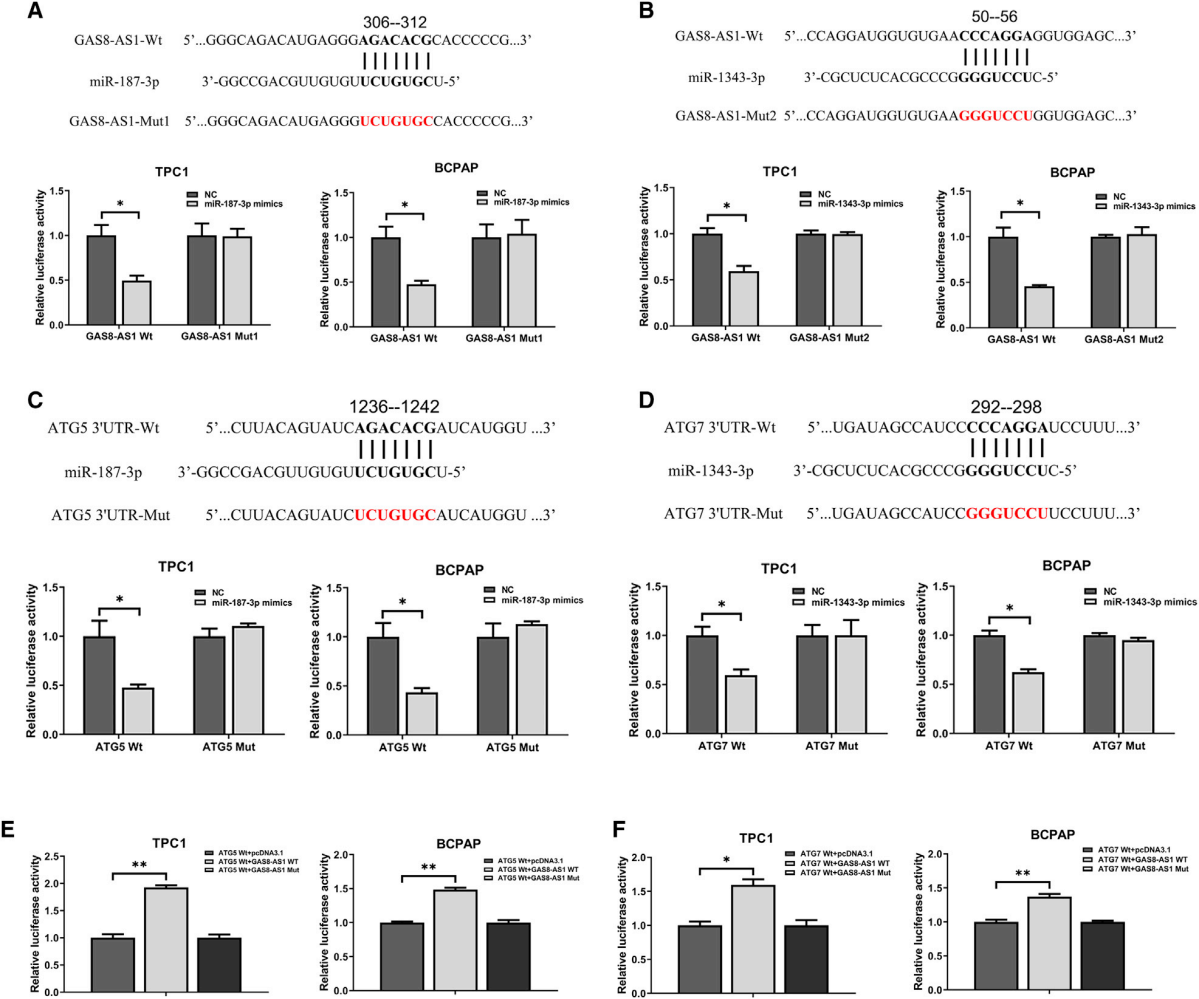
GAS8-AS1 Acts as a ceRNA, Regulating ATG5 and ATG7 Expression via miR-187-3p and miR-1343-3p Binding, Respectively (A and B) The predicted miR-187-3p (A) and miR-1343-3p (B) binding sites in GAS8-AS1 (GAS8-AS1-WT) and the designed mutant sequences (GAS8-AS1-Mut1, GAS8-AS1-Mut2). TPC1 and BCPAP cells were transfected with GAS8-AS1-WT, GAS8-AS1-Mut1, GAS8-AS1-Mut2, and the indicated miRNAs, and then the luciferase reporter assay was conducted. (C) The predicted miR-187-3p binding sites in the ATG5 3′UTR (ATG5 3′UTR-WT) and the designed mutant sequence (ATG5 3′UTR-Mut). (D) The predicted miR-1343-3p binding sites in the ATG7 3′UTR (ATG7 3′UTR-WT) and the designed mutant sequence (ATG7 3′UTR-Mut). TPC1 and BCPAP cells were transfected with ATG5 3′UTR-WT, ATG5 3′UTR-Mut, ATG7 3′UTR-WT, ATG7 3′UTR-Mut, and the indicated miRNAs, and then the luciferase reporter assay was conducted. (E) Luciferase reporter assay of cells co-transfected with ATG5 3′UTR-WT and the vectors pcDNA3.1, GAS8-AS1-WT, and GAS8-AS1-Mut1. (F) Luciferase reporter assay of cells co-transfected with ATG7 3′UTR-WT and the vectors pcDNA3.1, GAS8-AS1-WT, and GAS8-AS1-Mut2. Statistical differences were analyzed using the independent samples t test; data are shown as the mean ± standard error of the mean based on three independent experiments. ∗p < 0.05, ∗∗p < 0.01.

We next demonstrated whether GAS8-AS1 could function as a ceRNA by directly sponging miR-187-3p and miR-1343-3p. Bioinformatics prediction showed that miR-187-3p and miR-1343-3p could bind to GAS8-AS1 and directly target the 3′ UTRs of ATG5 and ATG7, respectively. The dual-luciferase reporter assays showed that upregulating miR-187-3p significantly reduced the luciferase activity of PTC cells co-transfected with wild-type GAS8-AS1 (GAS8-AS1-WT), while upregulating miR-187-3p had no effect on luciferase activity when the cells were co-transfected with mutated GAS8-AS1 (GAS8-AS1-Mut1) (Figure 6A). Co-transfection of miR-1343-3p mimic and GAS8-AS1-WT significantly reduced the luciferase activity, while luciferase activity was unchanged by miR-1343-3p mimic and GAS8-AS1-Mut2 co-transfection (Figure 6B), suggesting that GAS8-AS1 binds directly to miR-187-3p and miR-1343-3p. Similarly, the dual-luciferase reporter system was used to determine whether ATG5 and ATG7 are direct targets of miR-187-3p and miR-1343-3p, respectively. Compared with the NC group, miR-187-3p markedly inhibited ATG5-3′ UTR-WT luciferase activity, whereas that of the ATG5-3′ UTR-Mut was not notably affected (Figure 6C). The ectopic overexpression of miR-1343-3p significantly suppressed ATG7-3′ UTR-WT luciferase activity but failed to affect that of the ATG7-3′ UTR-Mut (Figure 6D). The results indicate that, in PTC cells, miR-187-3p and miR-1343-3p bind specifically to the ATG5 3′ UTR and ATG7 3′ UTR, respectively. After confirming that miR-187-3p could directly bind to both GAS8-AS1 and the ATG5 3′ UTR, and that miR-1343-3p could directly bind to both GAS8-AS1 and the ATG7 3′ UTR, another dual-luciferase reporter assay was applied to confirm whether GAS8-AS1 could regulate ATG5 and ATG7 by interacting with miR-187-3p and miR-1343-3p, respectively. The GAS8-AS1-WT significantly elevated the luciferase activity of the WT ATG5 and ATG7; however, the GAS8-AS1-Mut did not affect the luciferase activity of the WT ATG5 and ATG7 (Figures 6E and 6F). Taken together, our results indicate that GAS8-AS1 modulates ATG5 and ATG7 expression as a ceRNA by sponging miR-187-3p and miR-1343-3p.

In addition, studies have shown that patients with PTC have mutant types of GAS8-AS1, mainly a c.713A>G/714T>C dinucleotide substitution.^10^ To clarify whether the mutation would affect GAS8-AS1 regulation of miRNA, we constructed a mutant GAS8-AS1 plasmid (G713C714). The qRT-PCR results showed that, compared with WT GAS8-AS1, the mutant GAS8-AS1 could not regulate miR-187-3p and miR-1343-3p expression in the TPC1 and BCPAP cells (Figure S4).

### GAS8-AS1 Inhibited Tumor Growth *In Vivo*

**Figure 7:**
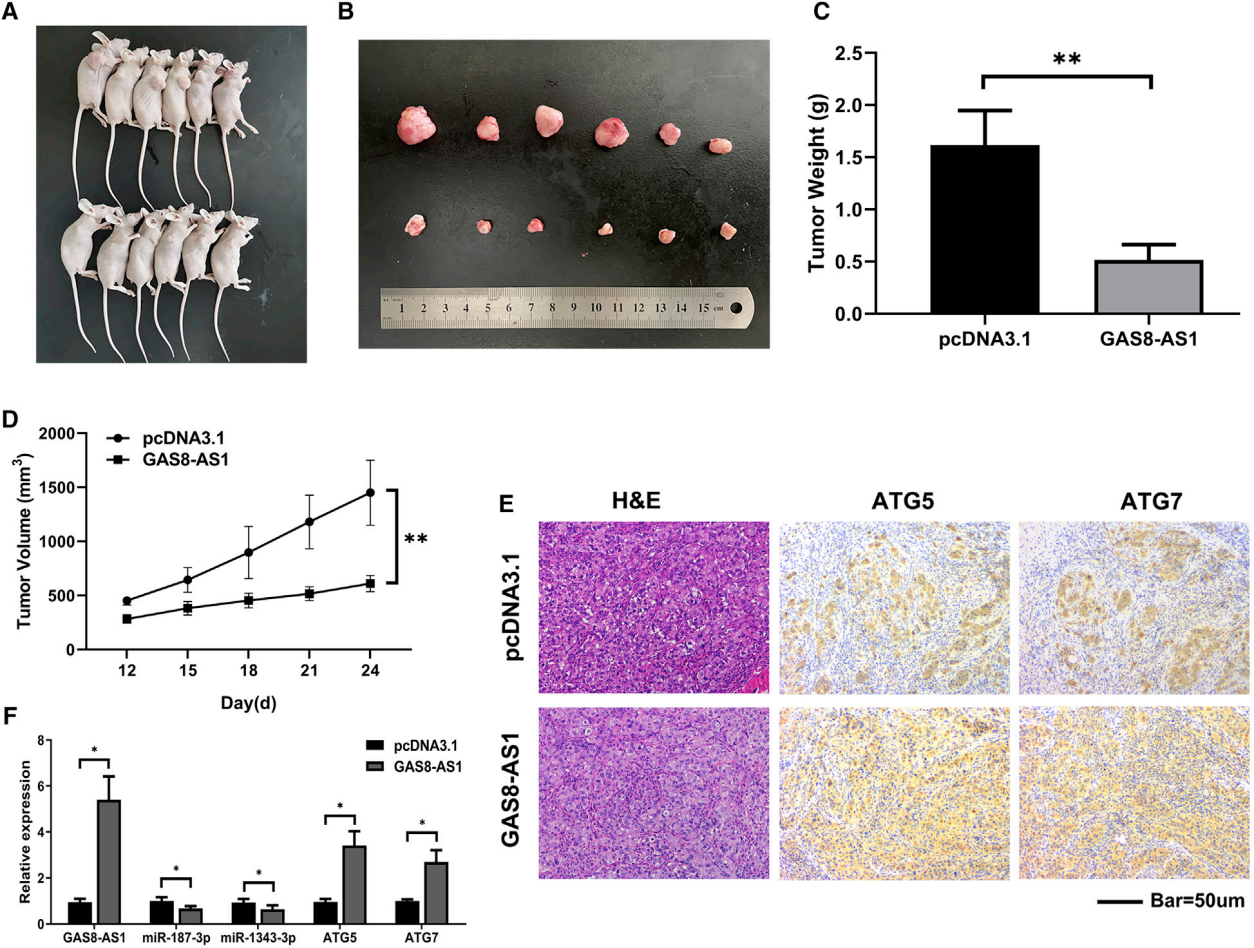
GAS8-AS1 Inhibited Tumor Growth *In Vivo* (A and B) The subcutaneous implant mouse model was established after inoculation with vectors pcDNA3.1 or pcDNA3.1-GAS8-AS1 TPC1 cells (n = 6). (C and D) Harvested tumor volumes (C) and tumor weights (D) were measured for both groups. (E) Tumor sections were stained with H&E (hematoxylin and eosin). ATG5 and ATG7 levels were examined by IHC. (F) qRT-PCR measurement of the relative expression of GAS8-AS1, miR-187-3p, miR-1343-3p, ATG5, and ATG7 in the two groups. Statistical differences were analyzed using the independent samples t test; data are shown as the mean ± standard error of the mean based on three independent experiments. ∗p < 0.05, ∗∗p < 0.01.

To clarify whether GAS8-AS1 influenced tumor growth *in vivo*, TPC1 cells stably transfected with GAS8-AS1 overexpression vector or control vector (pcDNA3.1) were injected into the backs of the mice. The growth of subcutaneous tumors was observed every 3 days from day 10 after injection; the long and short diameters of the tumors were determined using a Vernier caliper, and the tumor volume was calculated accordingly. Xenograft tumor assays indicated that the GAS8-AS1 overexpression xenograft tumors were smaller than that of the NC vector (Figures 7A and 7B). The tumor volumes and weights were markedly reduced in the GAS8-AS1 overexpression group compared with the NC vector group (Figures 7C and 7D). Consistent with the *in vitro* observations, the GAS8-AS1 overexpression group had upregulated levels of ATG5 and ATG7 protein (Figure 7E). Additionally, the excised tumor masses showed that GAS8-AS1 overexpression triggered an increase in GAS8-AS1, ATG5, and ATG7 and a reduction in miR-187-3p and miR-1343-3p (Figure 7F).

### ATF2 Induced GAS8-AS1 Expression by Functioning as a TF

**Figure 8:**
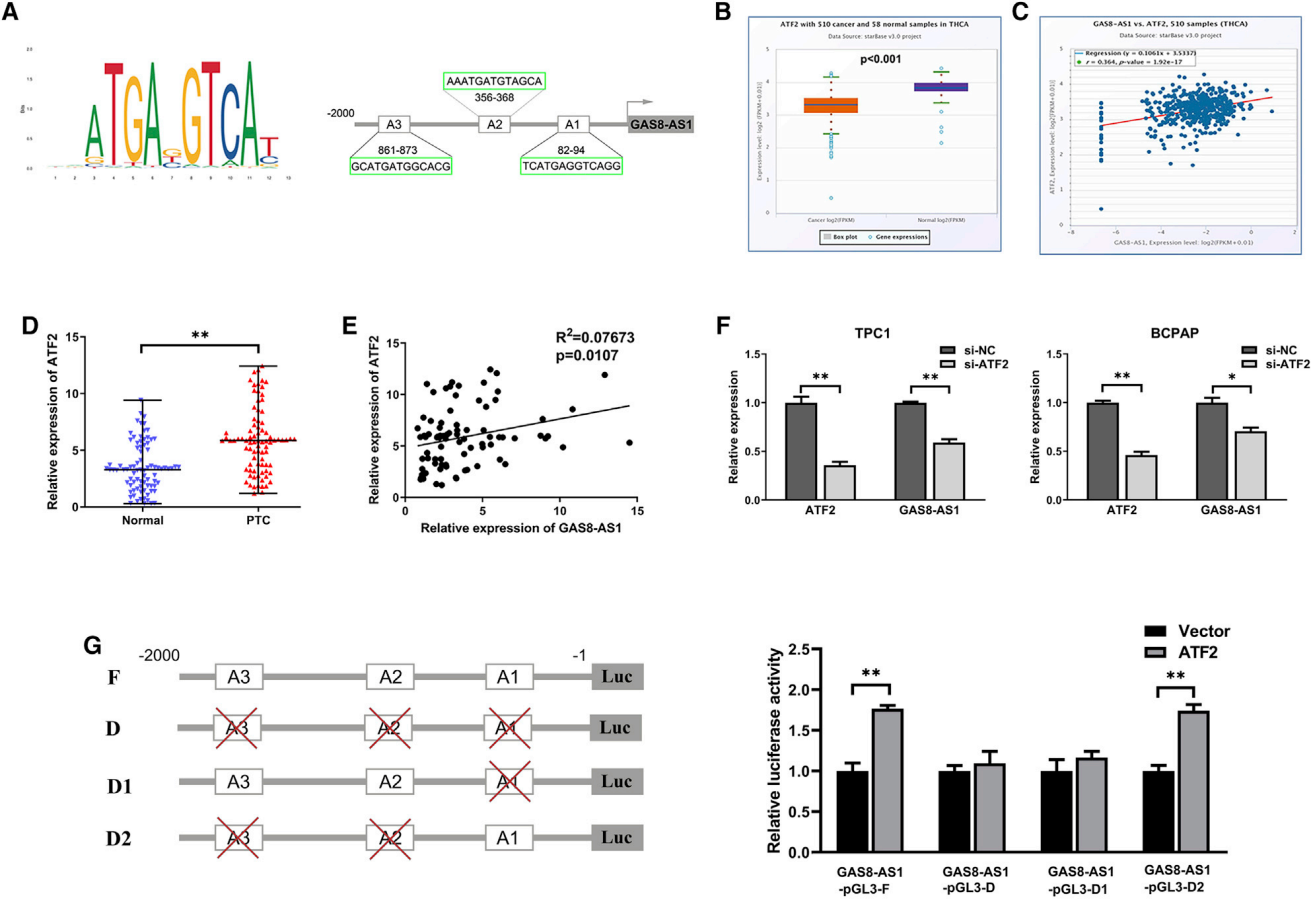
ATF2 Induced GAS8-AS1 Expression by Functioning as a TF (A) JASPAR predicted the positions of the putative ATF2 binding motif at −2,000 bp in the human GAS8-AS1 promoter. (B) GAS8-AS1 expression in PTC tissues and normal tissues from TCGA database. (C) Correlations between GAS8-AS1 and ATF2 in PTC tissues from TCGA database. (D) The relative expression level of ATF2 in 84 pairs of PTC tissues and adjacent non-cancerous tissues. Statistical differences were analyzed using the Wilcoxon signed-rank test; data are shown as the median and range. (E) Correlations between GAS8-AS1 and ATF2 in PTC tissues were analyzed using Pearson’s correlation. (F) The expression levels of GAS8-AS1 and ATF2 following si-ATF2 treatment in TPC1 and BCPAP cells. (G) Luciferase reporter assay performed following co-transfection of the full-length GAS8-AS1 promoter (GAS8-AS1-pGL3-F) or deleted GAS8-AS1 promoter fragments (GAS8-AS1-pGL3-D, GAS8-AS1-pGL3-D1, GAS8-AS1-pGL3-D2) with the ATF2 expression plasmid or blank vector in TPC1 cells. Luciferase activities were expressed as relative to that of the pGL3 vector. Statistical differences were analyzed using the independent samples t test; data are shown as the mean ± standard error of the mean based on three independent experiments. ∗p < 0.05, ∗∗p < 0.01.

**Figure 9:**
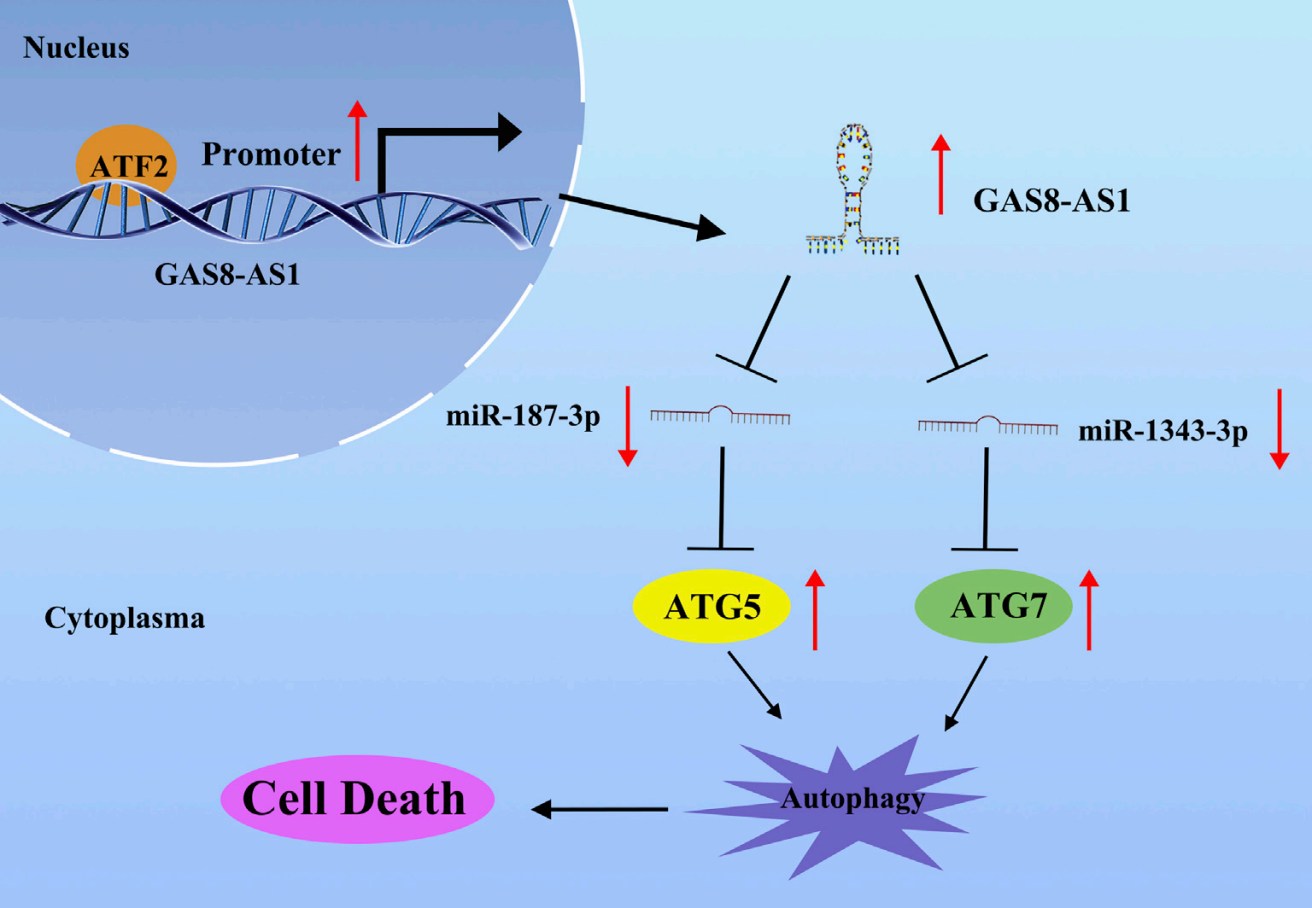
Summary of the Regulation and Mechanism of GAS8-AS1 in PTC Cells

There is accumulating evidence that several key TFs contribute to lncRNA dysregulation in human cancers. To analyze the TFs that activate GAS8-AS1, we used the UCSC Genome Browser (http://genome.ucsc.edu/) to find the 2,000-bp sequence upstream of the GAS8-AS1 start point, and JASPAR predicted a possible binding TF. ATF2 is an upstream TF that binds the GAS8-AS1 promoter at position 82–94 bp (Figure 8A) with a high score of 13.8 (Figure S5). TCGA database analysis revealed that ATF2 was downregulated in thyroid cancer tissue (Figure 8B) and that there was a positive correlation between ATF2 and GAS8-AS1 expression in PTC tissue (Figure 8C). The tissue samples in the present study yielded the same results (Figures 8D and 8E). Silencing ATF2 significantly decreased ATF2 and GAS8-AS1 expression in the PTC cells (Figure 8F). The dual-luciferase assay showed that ATF2 could bind to the A1 binding site of the GAS8-AS1 promoter region but not the other two sites (Figure 8G). The results showed that upregulation of ATF2 activated GAS8-AS1–promoted autophagy of PTC cells by sponging oncogenic miR-187-3p and miR-1343-3p and upregulating the expression of ATG5 and ATG7, respectively, thereby inhibiting the proliferation of PTC cells (Figure 9).

## Discussion

The physiological and pathological effects of lncRNAs are achieved primarily through gene expression regulation. Transcription and translation are key phases of these biological processes.^26^^,^^27^ lncRNAs are involved specifically in epigenetic regulation, TF regulation, post-transcriptional regulation, and protein degradation in cancer. They epigenetically regulate the expression of many salient genes involved in vital cellular biological processes, such as autophagy, cell differentiation, cell cycle regulation, cell proliferation, migration and invasion, apoptosis, and mesenchymal stem cell differentiation.^28^^,^^29^ Generally, lncRNAs exert their regulatory functions in various modes. For example, lncRNAs can act as a *cis* element to recruit or impede TFs and thereby modulate transcriptional productivity, function as genomic structural scaffolding to initiate protein complex assembly or as an miRNA sponge to regulate downstream gene expression, and encode a small peptide that influences cancer progression.^30^ lncRNAs have been widely reported in liver cancer, gastric cancer, colon cancer, and other tumors but remain in the initial stage in the study of thyroid cancer. The study of GAS8-AS1, a novel lncRNA, is still in its infancy. Pan et al.^10^ performed whole-exome sequencing of 91 clinically and pathologically characterized PTC tissues and 91 paired peripheral blood samples and found that GAS8-AS1 was the second most frequently altered gene and acts as a novel tumor suppressor in PTC. Subsequent research found that GAS8-AS1 suppresses hepatocarcinogenesis by epigenetically activating the tumor suppressor GAS8.^12^ Zhao et al.^11^ demonstrated that GAS8-AS1 inhibited cell proliferation in colorectal cancer by downregulating the lncRNA AFAP1-AS1. Here, we checked TCGA-THCA (TCGA thyroid cancer data collection) and found that GAS8-AS1 was significantly decreased in thyroid cancer tissues compared to normal non-tumor tissues. We also observed downregulated GAS8-AS1 expression in 84 PTC tissues. More importantly, lower relative expression of GAS8-AS1 was correlated with poorer prognosis, which included higher TNM stage and LNM, indicating the potential role of GAS8-AS1 in PTC pathopoiesis. Therefore, the antioncogenic role of GAS8-AS1 in PTC is well established.

Autophagy is a highly conservative process of cell self-degradation, which plays a vital role under stress conditions and cell survival.^31^ Dysregulation of autophagy function led to the two-way effect of promoting and inhibiting cancer processes.^32^^,^^33^ ATG5 and ATG7 have previously been reported to be the mutual targets of different lncRNAs for regulating autophagy in several tumor cells. The lncRNA ZNNT1 inhibits tumorigenesis of uveal melanoma by regulating the expression of the key autophagy gene ATG5.^34^ Chen et al.^35^ demonstrated that the lncRNA HULC functions as an oncogene by targeting ATG7 and ITGB1 in epithelial ovarian carcinoma. Previous experiments have confirmed that GAS8-AS1 can promote autophagy by upregulating ATG5 in PTC cells. To further investigate the downstream target proteins of GAS8-AS1, we focused on detecting the autophagy-associated genes and found that ATG5 and ATG7 were significantly decreased in the si-GAS8-AS1 group and were increased in the GAS8-AS1 overexpression group as compared with the NC group, at both the mRNA and protein levels. Tissue expression detection demonstrated that ATG5 and ATG7 were downexpressed in PTC tissues and were correlated positively with GAS8-AS1.

Emerging evidence suggests that lncRNAs might function as ceRNAs to sponge miRNAs via sequence complementarity and subsequently influence the functional roles of miRNAs.^36^ For example, LINC01133 inhibits gastric cancer progression by sponging miR-106a-3p to regulate APC expression and the Wnt–β-catenin pathway.^37^ The lncRNA C5orf66-AS1 promoted cell proliferation in cervical cancer by targeting the miR-637/RING1 axis.^38^ Here, we found that GAS8-AS1 mainly localized in the cytoplasm of PTC cells. Therefore, we speculated that GAS8-AS1 might also function as a ceRNA, regulating ATG5 and ATG7 expression by sponging miRNAs in PTC. Bioinformatics analysis and target prediction tools identified miRNAs that could not only target ATG5 or ATG7 but also have binding sites for GAS8-AS1. Initially, 16 miRNAs were predicted to interact with both GAS8-AS1 and the 3′ UTR of ATG5 or ATG7. miRNA expression in PTC cells with GAS8-AS1 upregulation or downregulation was detected using qRT-PCR, and we found that miR-187-3p, miR-216a-5p, miR-449a, and miR-1343-3p might be candidate miRNAs. Western blotting indicated that only miR-187-3p and miR-1343-3p could downregulate ATG5 and ATG7 levels, respectively. Therefore, we speculated that ATG5 is the target gene of miR-187-3p and that ATG7 is the target gene of miR-1343-3p.

Subsequently, we found that miR-187-3p and miR-1343-3p were significantly overregulated in the 84 pairs of PTC tissues and matched normal tissues. The correlation analysis showed that GAS8-AS1 levels correlated negatively with those of miR-187-3p and miR-1343-3p. The functional experiments indicated that miR-187-3p and miR-1343-3p could promote proliferation and inhibit the activation of autophagy in PTC cells. Finally, we used a rescue strategy to confirm that miR-187-3p and miR-1343-3p could restore the proliferation and autophagy effect of GAS8-AS1 on PTC cells. Dual-luciferase reporter assays were performed in PTC cells to confirm that miR-187-3p could bind directly to both GAS8-AS1 and the ATG5 3′ UTR and that miR-1343-3p could bind directly to both GAS8-AS1 and the ATG7 3′ UTR. The results suggested that miR-187-3p is the vital miRNA that binds to both GAS8-AS1 and the ATG5 3′ UTR, and that miR-1343-3p is the critical miRNA that binds to both GAS8-AS1 and the ATG7 3′ UTR, indicating that GAS8-AS1 promotes PTC cell autophagy through the miR-187-3p/ATG5 axis and the miR-1343-3p/ATG7 axis, respectively.

Patients with PTC have multiple types of GAS8-AS1 mutation, mainly a c.713A>G/714T>C dinucleotide substitution, accounting for about 90% of all mutant GAS8-AS1.^10^ Mutant GAS8-AS1 (G713C714) can also inhibit PTC cell proliferation, although the inhibitory effect is weaker than that of the WT GAS8-AS1.^10^ As the mutation site is not at the binding site of GAS8-AS1 with miR-187-3p and miR-1343-3p, we speculated whether the mutant GAS8-AS1 (G713C714) would also bind to the two miRNAs and then function. Therefore, we constructed TPC1 and BCPAP cells overexpressing mutant and WT GAS8-AS1 and then detected the expression levels of miR-187-3p and miR-1343-3p using qRT-PCR. Compared with the control group, miR-187-3p and miR-1343-3p expression levels in PTC cells were downregulated in the WT GAS8-AS1 overexpression group, but there was no change in the mutant GAS8-AS1 overexpression group, indicating that the mutant GAS8-AS1 could not regulate miR-187-3p and miR-1343-3p expression. The secondary structure of lncRNA affects the binding of a miRNA to its specific miRNA recognition elements (MREs).^39^ For example, a change in the secondary structure of MALAT1 disrupted miR-217-5p binding to MALAT1.^40^ Pan et al.^10^ demonstrated that the c.713A>G/714T>C dinucleotide substitution induces a significant change in the GAS8-AS1 secondary structure, so we speculated that the difference of the secondary structure between the mutant and WT GAS8-AS1 may have caused this result.

There is evidence indicating that TFs play an important role in lncRNA dysregulation. TFs regulate lncRNA expression by binding specifically to the promoter region of lncRNAs, as with some protein-coding genes. For example, the TF SP1 activates transcription of the lncRNA AGAP2-AS1 in breast cancer.^41^ The FOXO1-regulated lncRNA LINC01197 inhibits pancreatic adenocarcinoma cell proliferation.^42^ Therefore, we searched for a TF that might be linked to GAS8-AS1 dysregulation. ATF2 is a member of the activator protein 1 (AP-1) TF family of the basic leucine zipper (bZIP)-containing DNA-binding proteins and plays crucial roles in cellular development and survival.^43^ As a TF, ATF2 regulates miR-132 and miR-320a transcription by binding to their promoters.^44^^,^^45^ However, it has not been reported whether ATF2 can activate lncRNA transcription by binding to the lncRNA promoter. Here, the online bioinformatics analysis tool JASPAR predicted the binding sequence of ATF2 to the GAS8-AS1 promoter region. The results showed that three regions may be combined with ATF2 at 2,000 bp upstream of the GAS8-AS1 promoter, among which the A1 site had the highest score for binding ability. Subsequently, the results of TCGA database and tissue specimen validation confirmed that relative ATF2 expression was lower and correlated positively with GAS8-AS1 in PTC tissues. Finally, the dual-luciferase assay was used to verify the binding of ATF2 and the GAS8-AS1 promoter region. Four luciferase reporter genes were constructed and included the presence of the full-length promoter (non-deletion of possible binding sites); A1 site deletion; A2 and A3 site deletion; and A1, A2, and A3 site deletion. As expected, the luciferase activity after ATF2 overexpression in the non-deletion group and the A2 + A3 deletion group increased significantly compared with the other two groups, indicating that ATF2 bound to the GAS8-AS1 promoter region (A1 site) and activated GAS8-AS1 expression.

Based on the above experimental results, we conclude that the upregulation of ATF2 activated GAS8-AS1–promoted autophagy of PTC cells via the sponging of oncogenic miR-187-3p and miR-1343-3p and upregulated the expression levels of ATG5 and ATG7, respectively. Therefore, GAS8-AS1 could be a promising biomarker and therapeutic target in PTC.

## Materials and Methods

### Sample Collection

Eighty-four pairs of PTC tissues and the adjacent non-cancerous tissues were obtained from patients undergoing surgery at the First Hospital of China Medical University between 2014 and 2016. All samples were immediately dissected, placed on ice, snap-frozen in liquid nitrogen, and stored at −80°C until used. The patient tissue samples consisted of PTC tissues and the adjacent non-cancerous tissues, which were all confirmed by histopathological examination. Due to the limitation of the size of the thyroid, the adjacent non-cancerous tissues were normal thyroid tissues located >2 cm away from the tumor margins on the same lobe or from the opposite lobe. The collected clinicopathological characteristics included age, gender, extrathyroidal extension, TNM stage, LNM, multicentricity, tumor size, and Hashimoto thyroiditis. None of the patients had received preoperative local or systemic treatment. All procedures performed in studies involving human participants were in accordance with the ethical standards of the Research Ethics Committee of The First Hospital of China Medical University and with the 1964 Helsinki declaration and its later amendments. All written informed consent to participate in the study and for samples to be collected from them was obtained from patients with PTC.

### Cell Culture and Transfection

In our previous article, GAS8-AS1 was significantly downregulated in the PTC cell lines TPC1 and BCPAP and could inhibit cell proliferation and promote autophagy. Therefore, we continued to use the two cell lines for further study. The BCPAP cell line was from DSMZ (Braunschweig, Germany); the TPC1 cells were a gift from Professor Meiping Shen (Department of General Surgery, The First Affiliated Hospital of Nanjing Medical University, Nanjing, China). Cell culture medium was prepared according to the suppliers’ instructions. The BCPAP cells were maintained in RPMI 1640 (HyClone, Logan, UT, USA) supplemented with 10% fetal bovine serum (FBS; PAN-Biotech, Aidenbach, Germany). The TPC1 cells were maintained in high-glucose Dulbecco’s modified Eagle’s medium (DMEM, HyClone) supplemented with 10% FBS. All cells were cultured at 37°C in a humidified atmosphere with 5% CO_2_.

The expression plasmids containing GAS8-AS1 complementary DNA were from Obio Technology (Shanghai, China). siRNA and the negative control (NC) were from GenePharma (Suzhou, China). The PTC cells were transfected using Lipofectamine 3000 (Invitrogen, Waltham, MA, USA) according to the manufacturer’s protocol. The siRNA sequences are available in Table S1.

### Total RNA Isolation and qRT-PCR

Total RNA was isolated from frozen specimens collected from the tissue samples and cells using RNAiso (Takara, Dalian, China). A reverse transcription kit (RR036A, Takara, Shiga, Japan) was used to transcribe total RNA and produce complementary DNA. Gene expression was analyzed by qRT-PCR, which was performed with SYBR Premix Ex TaqII (Takara) and a LightCycler 480 system (Roche, Indianapolis, IN, USA). The amplification conditions were as follows: 95°C for 30 s, followed by 50 cycles of 95°C for 5 s and 60°C for 30 s; dissociation at 95°C for 60 s, 55°C for 1 min, and 95°C for 30 s. The miRNA antisense primer was included in the 638313 Mir-X miRNA First-Strand Synthesis Kit (Takara). The relative expression levels were calculated using the 2^−ΔCT^ method (cycle threshold [CT]). ΔCT indicated the difference in the CT value between the target and endogenous reference. GAPDH and U6 were used as internal controls. Each qRT-PCR was performed in triplicate to verify the stability and repeatability of the results. The primer sequences are available in Table S1.

### Subcellular Fractionation

The cytosolic and nuclear fractions of the TPC1 or BCPAP cells were separately isolated using a Cytoplasmic and Nuclear RNA Purification Kit (Norgen Biotek, ON, Canada) according to the manufacturer’s instructions. Briefly, the cell suspension was centrifuged and precipitated, lysis buffer J was added to split, and it was centrifuged. The remaining supernatant contained cytoplasmic RNA, which precipitated into nuclear RNA. The supernatant was transferred to an RNase-free tube. Buffer SK and 96%–100% ethanol were added to the cytoplasmic RNA fraction and nuclear RNA fraction, respectively. The mixture was applied to a spin column comprising a collection tube and centrifuge, the flow-through was discarded, and the spin column was reassembled with its collection tube. Subsequently, wash solution A was applied to the column and centrifuged, and then the flow-through was discarded. The previous step was repeated to wash the column three times. Elution buffer E (50 μL) was added to the column and centrifuged, and the remaining liquid contained the cytoplasmic RNA and nuclear RNA. The purified RNA sample could be stored at −20°C for a few days. It was recommended that samples be placed at −70°C for long-term storage.

### FISH

A probe was used to identify GAS8-AS1 rearrangement in the TPC1 and BCPAP cells (green-labeled; Boster, Wuhan, China) according to the manufacturer’s instructions. Briefly, the cells were seeded on 24-well slides and fixed with 4% paraformaldehyde (POM) for 30 min. The pepsin was digested with 3% citric acid for 2 min, then fixed in 1% POM for 30 min. The pre-hybridizing liquid was added at 38°C–42°C for 2–4 h, and then the hybridizing liquid was added at 38°C–42°C for overnight treatment. On the second day, the buffer was washed away, and the cells were treated with blocking solution for 30 min, biotinylated rat anti-digoxin for 2 h, SABC (streptavidin-biotin complex) for 30 min, and biotinylated peroxidase for 30 min. All fluorescence images were captured using a confocal microscope (Leica, Wetzlar, Germany).

### Cell Proliferation Assay

The proliferation rate of TPC1 and BCPAP cells that had been transfected with plasmids or siRNAs was determined using CCK-8 (Dojindo, Kumamoto, Japan). Briefly, 2 × 10^3^ cells per well were seeded in a 96-well plate in a final volume of 100 μL and were transfected with plasmids or siRNAs. Proliferation was evaluated at 0 h, 24 h, 48 h, 72 h, and 96 h after transfection, and the cells were incubated for 3 h at 37°C after 10 μL CCK-8 solution had been added to each well. The number of viable cells was calculated based on the absorbance at 450 nm.

### Western Blotting

Total proteins derived from the cells were extracted in lysis buffer, and the protein concentration was detected using a bicinchoninic acid protein assay kit (Beyotime, China), according to the manufacturer’s instructions. Proteins were fractionated by 10% or 12% sodium dodecyl sulfate-polyacrylamide gel electrophoresis. The separated proteins were transferred to polyvinylidene fluoride membranes (Bio-Rad, Hercules, CA, USA). The membranes were blocked in 5% skim milk for 2 h at room temperature and then incubated with primary antibodies at 4°C overnight. Next, the blotted membranes were incubated with horseradish peroxidase-conjugated anti-rabbit immunoglobulin G (1:20,000) secondary antibody at room temperature for 2 h. The proteins were visualized using an enhanced chemiluminescence detection system. Information on the primary antibodies is available in Table S2.

### IF

Cells (2 × 10^4^ cells/well) were seeded in 24-well plates. The cells were washed with phosphate-buffered saline after transfection and then fixed with 4% polyformaldehyde. The fixed cells were incubated with primary antibody against LC3 at 4°C overnight. After washing, a fluorescent secondary antibody was added and incubated for 2 h. Finally, the cells were counterstained with diaminophenylindole (DAPI, Beyotime) and visualized using a confocal microscope (Leica). Information on the antibodies is available in Table S2.

### TEM

The cells were collected with a cell scraper, centrifuged, and fixed in 2.5% glutaraldehyde in 0.1 M sodium cacodylate buffer. The cells were then dehydrated in a gradient of 50%–100% ethanol and embedded in araldite. Ultrathin sections were obtained (50–60 nm) and stained with uranyl acetate and lead citrate. A JEM-1400 transmission electron microscope was used at 80 kV, applying × 20,000 magnification.

### Luciferase Assay

GAS8-AS1 fragments containing the putative binding sites for miR-187-3p and miR-1343-3p were PCR-amplified and cloned in the firefly luciferase expression vector pmiR-REPORT (Obio Technology) and designated GAS8-AS1-WT. To mutate the miR-187-3p and miR-1343-3p putative binding sites in GAS8-AS1, the putative binding site sequences were replaced and were designated GAS8-AS1-Mut1/Mut2. The cells were seeded in 96-well plates the day before transfection and transfected with pmiR-REPORT-GAS8-AS1-WT, and the pmiR-REPORT-GAS8-AS1-Mut1/Mut2 reporter vectors, together with the Renilla luciferase-expressing vector pRL-TK (Promega, Madison, WI, USA) and miR-187-3p mimic, miR-1343-3p mimic, or NC using Lipofectamine 3000 (Invitrogen). Similarly, ATG5-3′UTR-WT and ATG5-3′UTR-Mut, containing the putative binding site of miR-187-3p, and ATG7-3′UTR-WT and ATG7-3′UTR-Mut, containing the putative binding site of miR-1343-3p, were established and cloned into the firefly luciferase expression vector pmiR-REPORT (Obio Technology). Again, the cells were seeded in 96-well plates the day before transfection and transfected with either pmiR-REPORT-ATG5-3′UTR-WT and the pmiR-REPORT-ATG5-3′UTR-Mut reporter vector or pmiR-REPORT-ATG7-3′UTR-WT and the pmiR-REPORT-ATG7-3′UTR-Mut reporter vector together with the Renilla luciferase expression vector pRL-TK and miR-187-3p mimic, miR-1343-3p mimic, or NC using Lipofectamine 3000. After 48 h, the cells were harvested, and the firefly and Renilla luciferase activity was measured using a dual-luciferase reporter assay system (Promega).

The ATF2 binding motif in the GAS8-AS1 promoter region was identified using JASPAR (http://jaspar.genereg.net/). The fragment sequences were synthesized and inserted into a pGL3-basic vector (Promega). All vectors were verified by sequencing, and luciferase activity was assessed using a dual-luciferase assay kit (Promega).

### IHC

We obtained fresh tumor tissue and the corresponding non-cancerous tissue from 84 patients with PTC to construct the tissue microarray (TMA). TMA sections were baked at 65°C for 30 min, then deparaffinized and rehydrated with xylene and alcohol gradients, respectively. Endogenous peroxidase activity was blocked with 3% H_2_O_2_. Then, the sections were treated with citrate buffer and microwave heated for 20 min before overnight incubation at 4°C with anti-ATG5 and anti-ATG7 antibody (1:200; Abcam, Cambridge, MA, USA). The immunohistochemical staining score was evaluated using the semi-quantitative Remmele scoring system.

### Xenograft Tumor Model

The xenograft tumor model was constructed in BALB/c nude mice (4–5 weeks old) purchased from Beijing Vital River Laboratory Animal Technology (Beijing, China). Tumor growth was monitored every 3 days; tumor volumes were estimated by length and width. After about 1 month, the mice were sacrificed, and the tumors were excised and weighed. All animal studies were conducted in accordance with the principles and procedures outlined in the guidelines of the Institutional Animal Care and Use Committee (IACUC) of China Medical University (IACUC approval number: TZ2019137).

### Statistical Analysis

All statistical analyses were performed with SPSS 22.0 (IBM, Chicago, IL, USA) and GraphPad Prism 8.0 (GraphPad Software, La Jolla, CA, USA). The relative expression of GAS8-AS1, miR-187-3p, miR-1343-3p, ATG5, and ATG7 in the PTC tissues and adjacent non-cancerous tissues were analyzed using the Wilcoxon signed-rank test. The correlation between GAS8-AS1 expression and clinicopathological characteristics was examined using chi-square. Data are presented as mean ± standard deviation (SD), and statistical analyses were performed using Student’s t test or analysis of variance. The differences were deemed statistically significant at ∗p < 0.05 and ∗∗p < 0.01.

## Author Contributions

Y.Q. and W.S. designed this study, performed the statistical analysis, and drafted the manuscript; Z.W. and W.D. analyzed the data; L.H. and T.Z. critically revised the work for important intellectual content; L.S. collected tissue samples; and H.Z. supervised the study. All authors read and gave final approval of the manuscript.

## Conflicts of Interest

The authors declare no competing interests.

## Supplemental Information

Document S1. Figures S1–S5 and Tables S1 and S2

Document S2. Article plus Supplemental Information
