# Supplementary material for: ATF2-Induced lncRNA GAS8-AS1 Promotes Autophagy of Thyroid Cancer Cells by Targeting the miR-187-3p/ATG5 and miR-1343-3p/ATG7 Axes
Source: Mol Ther Nucleic Acids. 2020 Sep 23;22:584–600. doi: 10.1016/j.omtn.2020.09.022 (PMC7562962; doi:10.1016/j.omtn.2020.09.022)
Supplement: Document S1. Figures S1–S5 and Tables S1 and S2 [file mmc1.pdf]

OMTN, Volume *22*

Supplemental Information

ATF2-Induced lncRNA GAS8-AS1 Promotes Autophagy of Thyroid Cancer Cells by Targeting the miR-187-3p/ATG5 and miR-1343-3p/ATG7 Axes

Yuan Qin, Wei Sun, Zhihong Wang, Wenwu Dong, Liang He, Ting Zhang, Liang Shao, and Hao Zhang

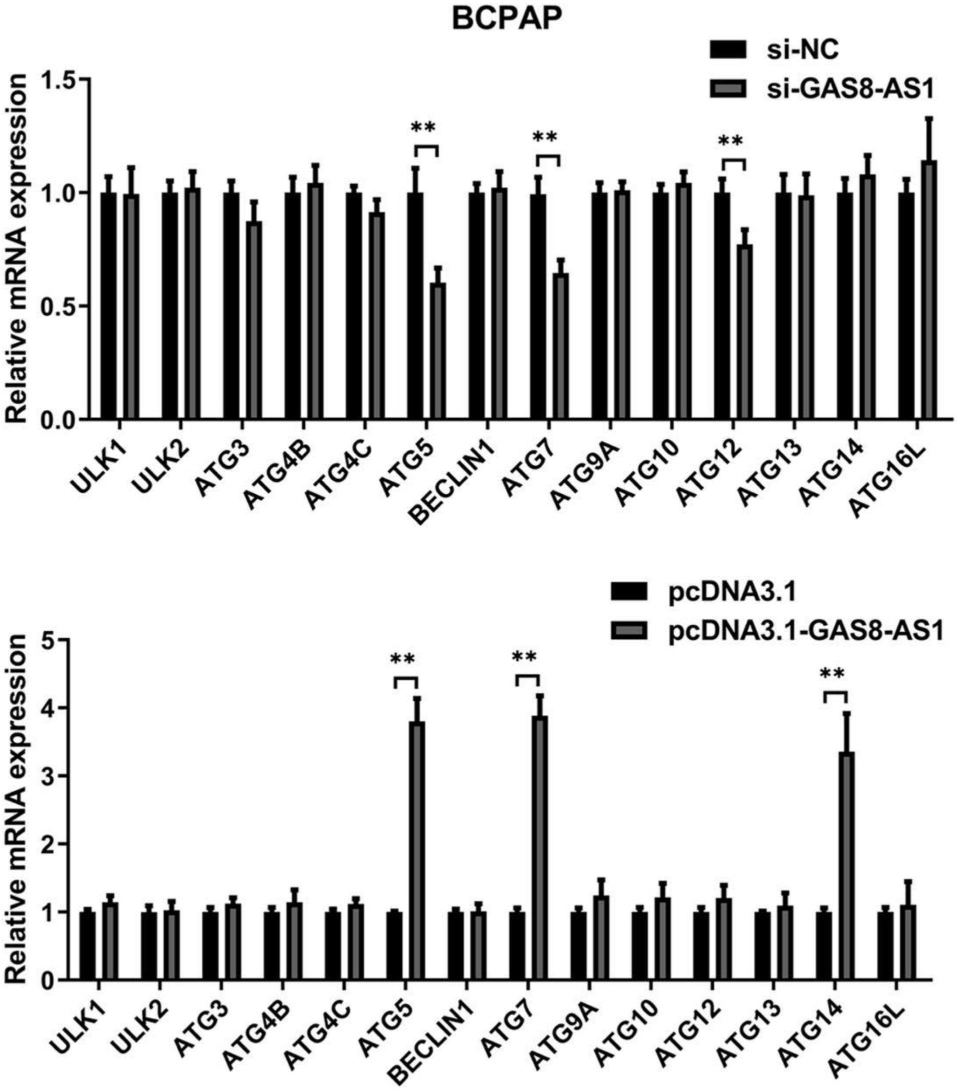

Figure S1. The relative expression level of related ATGs after down-regulated and up-regulated GAS8-AS1 were detected by qRT-PCR in BCPAP cells. Statistical differences were analyzed using the independent samples t-test; data are shown as the mean ± standard error of the mean based on three independent experiments. *$p < 0.05$, **$p < 0.01$.

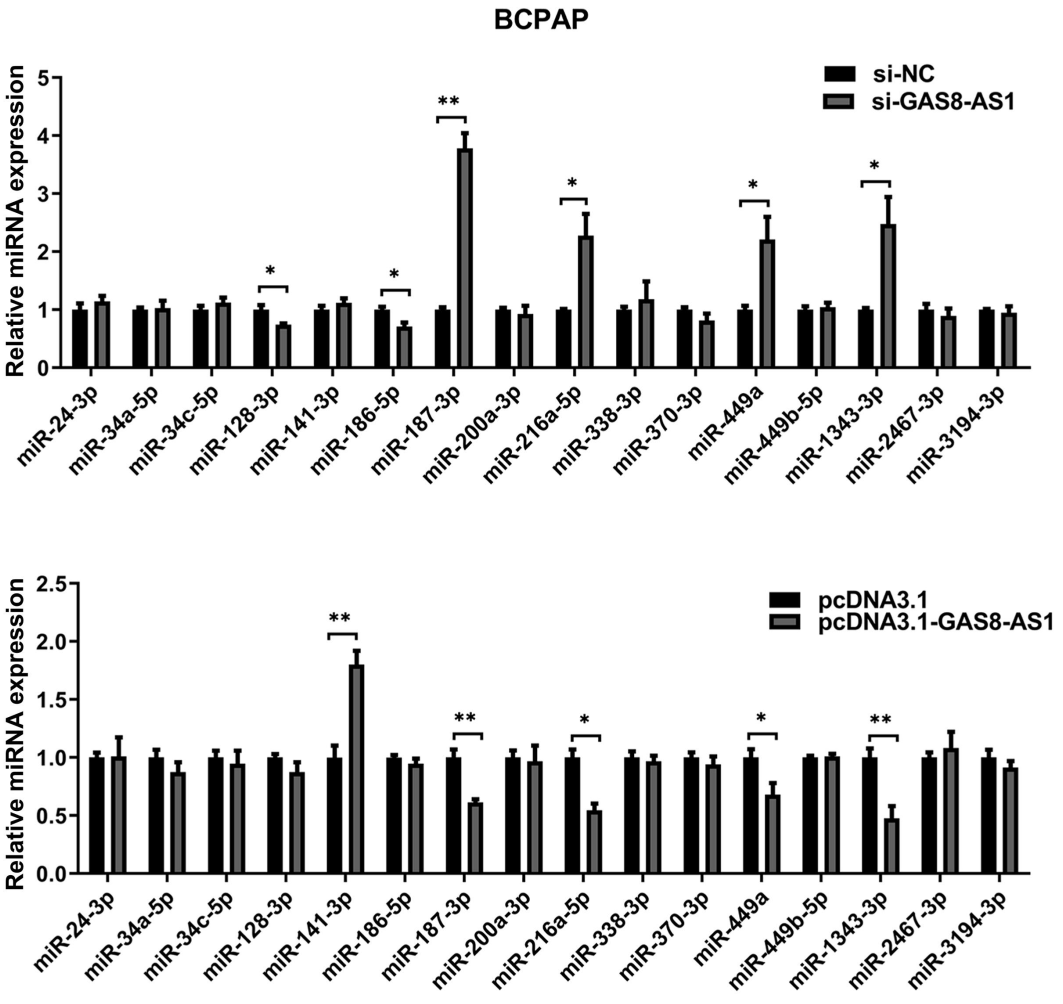

Figure S2. The relative expression level of the predicted miRNAs after down-regulated and up-regulated GAS8-AS1 were detected by qRT-PCR in BCPAP cells. Statistical differences were analyzed using the independent samples t-test; data are shown as the mean ± standard error of the mean based on three independent experiments. *$p < 0.05$, **$p < 0.01$.

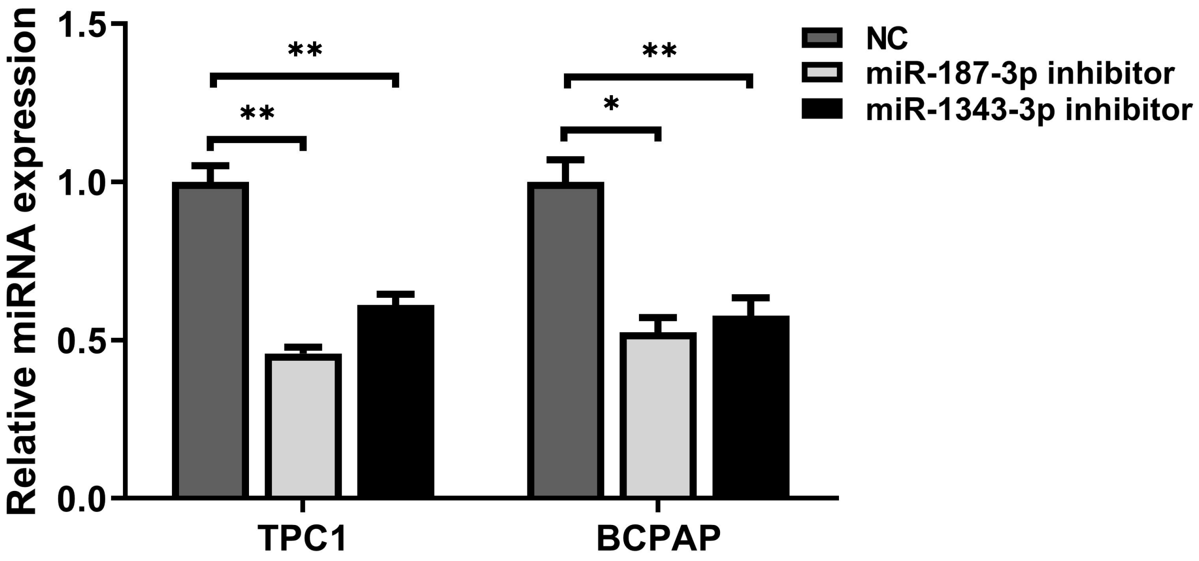

Figure S3. qRT-PCR analysis of interference efficiency after miR-187-3p and miR-1343-3p inhibitor transfection in PTC cells. Statistical differences were analyzed using the independent samples t-test; data are shown as the mean ± standard error of the mean based on three independent experiments. *p < 0.05, **p < 0.01.

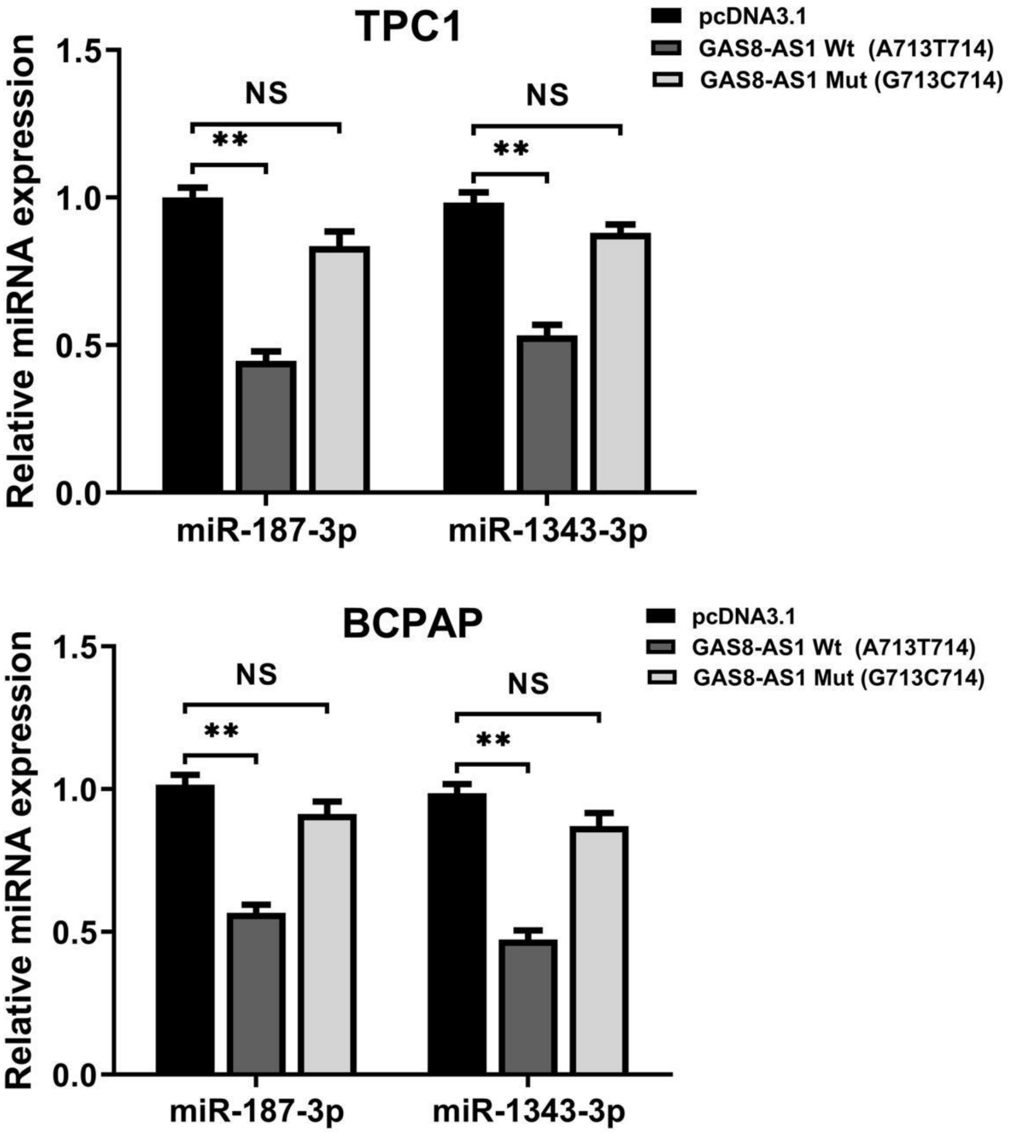

Figure S4. The relative expression of miR-187-3p and miR-1343-3p after transfection of the wild-type GAS8-AS1 (A713T714) expression vector, mutant GAS8-AS1 (G713C714) expression vector and control vector in TPC1 and BCPAP cells. Statistical differences were analyzed using the independent samples t-test; data are shown as the mean ± standard error of the mean based on three independent experiments. $*p < 0.05$, $**p < 0.01$.

Matrix ID	Name	Score	Relative score	Sequence ID	Start	End	Strand
MA1632.1	ATF2	13.8078	0.932736695899	hg38_knownGene_ENST00000408886.4	1906	1918	+
MA1632.1	ATF2	6.82297	0.826635329012	hg38_knownGene_ENST00000408886.4	1127	1139	+
MA1632.1	ATF2	6.38939	0.820049263362	hg38_knownGene_ENST00000408886.4	1632	1644	+

Figure S5. The predicted binding score between ATF2 and the GAS8-AS1 promoter.

Table S1. Primers and siRNA sequences used in this paper

Primer	Sense (5′–3′)	Antisense (5′–3′)
GAS8-AS1	GACAAGACAACGAGCAAACAAG	GGAGCCTCTAAAGGTCTGTGAC
ULK1	GGAATTCAAGCGTTCCTCTGGG	GCTCTTGTGCAAAGCAGCCTCAC
ULK2	CACAGAACGACCAATGGATG	TTGTTCGAAGGACCATGTGA
ATG3	GACCCCGGTCCTCAAGGAA	TGTAGCCCATTGCCATGTTGG
ATG4B	ATGGGAGTTGGCGAAGGCAAGT	AGCTCCACGTATCGAAGACAGC
ATG4C	TATTGGTGGCAAACCTAAACAGTC	TTGGGAGAAGGGCAGTGGAATGA
ATG5	AATCAGGTTTGGTGGAGGCA	CAGTGGAGGAAAGCAGAGGTG
Beclin1	GGCGGCTCCTATTCCATC	CCCAAGCAAGACCCCACT
ATG7	CGTTGCCCACAGCATCATCTTC	CACTGAGGTTCACCATCCTTGG
ATG9A	GCTTCCTCAAGGAGCAGGTTCA	CCACATTTGCGATAAGGCTCAGG
ATG10	CCCAGCAGGAACATCCAATA	AGGCTCAGCCATGATGTGAT
ATG12	TGACCTGCTGGCTGAATACC	GTGAAACCAAAACGCCTAACC
ATG13	CAGAACTGCTGGTGAGGACACT	AGCAGGCTGATAGGAAAGGCGA
ATG14	AAAGACGGGTGTGAGAGACC	GGTGTCTCCGTTGTGATCGT
ATG16L1	CTACGGAAGAGAACCAGGAGCT	CTGGTAGAGGTTCCTTTGCTGC
GAPDH	GCACCGTCAAGGCTGAGAAC	TGGTGAAGACGCCAGTGGA
miR-24-3p*	TGGCTCAGTTCAGCAGGAACAG	
miR-34a-5p	TGGCAGTGTCTTAGCTGGTTGT	
miR-34c-5p	AGGCAGTGTAGTTAGCTGATTGC	
miR-128-3p	TCACAGTGAACCGGTCTCTTT	
miR-141-3p	TAACACTGTCTGGTAAAGATGG	
miR-186-5p	CAAAGAATTCTCCTTTTGGGCT	
miR-187-3p	TCGTGTCTTGTGTTGCAGCCGG	
miR-200a-3p	TAACACTGTCTGGTAACGATGT	
miR-216a-5p	TAATCTCAGCTGGCAACTGTGA	
miR-338-3p	TCCAGCATCAGTGATTTTGTTG	
miR-370-3p	GCCTGCTGGGGTGGAACCTGGT	
miR-449a	TGGCAGTGTATTGTTAGCTGGT	
miR-449b-3p	CAGCCACAACTACCCTGCCACT	
miR-1343-3p	CTCCTGGGGCCCGCACTCTCGC	
miR-2467-3p	AGCAGAGGCAGAGAGGCTCAGG	
miR-3194-3p	AGCTCTGCTGCTCACTGGCAGT	
U6	CTCGCTTCGGCAGCACA	AACGCTTCACGAATTTGCGT
**siRNA**	**Sense (5′–3′)**	**Antisense (5′–3′)**
GAS8-AS1 siRNA	GGCACAACGACAAATGTCTTT	AGACATTTGTCGTTGTGCCTT
ATG5 siRNA	GGAATATCCT GCAGAAGAAT T	TTCTTCTGCA GGATATTCCT T

ATG7 siRNA	GAAGATAACAATTGGTGTATT	
ATF2 siRNA	GCTTCAGAAGATGACATTA	

*The antisense primer for the miRNA was included in the 638313 Mir-X™ miRNA First-Strand Synthesis Kit (Takara)

Table S2. Information on the antibodies used in this paper

Antibody	Brand	Concentration
ATG5	Abcam, ab108327	1:2500 (WB)
ATG7	Abcam, ab133528	1:2500 (WB)
LC3	Abcam, ab192890	1:2000 (WB)
p62	Abcam, ab109012	1:2000 (WB)
GAPDH	Proteintech, 10494-1-AP	1:1000 (WB)
ATG5	Abcam, ab108327	1:200 (IF)
ATG7	Abcam, ab133528	1:200 (IF)
LC3	Abcam, ab192890	1:200 (IF)
